# Transcriptomic and Metabolic Analysis Reveal Potential Mechanism of Starch Accumulation in *Spirodela polyrhiza* Under Nutrient Stress

**DOI:** 10.3390/plants14111617

**Published:** 2025-05-26

**Authors:** Xin Fang, Yan Hong, Yang Fang, Li Cheng, Zhaofeng Li, Caiming Li, Xiaofeng Ban

**Affiliations:** 1 State Key Laboratory of Food Science and Resources, Jiangnan University, Wuxi 214122, China; fangxin2226@163.com (X.F.); chenglichocolate@163.com (L.C.); zfli@jiangnan.edu.cn (Z.L.); licaiming2009@126.com (C.L.); banxiaofeng@jiangnan.edu.cn (X.B.); 2 Jiaxing Institute of Future Food, Jiaxing 314050, China; 3 School of Food Science and Technology, Jiangnan University, Wuxi 214122, China; 4 Key Laboratory of Environmental and Applied Microbiology, Chengdu Institute of Biology, Chinese Academy of Sciences, Chengdu 610041, China

**Keywords:** duckweed, starch, nutrient stress, starch accumulation, photosynthesis, starch enzyme, transcriptomic analyses

## Abstract

Compared with traditional grain starch sources, duckweed (*Spirodela polyrhiza*, *S. polyrhiza* for simple) does not require soil to produce starch, and the process is less affected by the external environment. Moreover, it produces high levels of starch under certain conditions. This study investigated the patterns and mechanisms of starch accumulation in *S. polyrhiza ZH0196* under nutrient stress by determining the changes in starch content, photosynthesis, and amylase activity at different stress induction times. Under nutrient stress, the culture solution was replaced with deionized water. The starch content increased from 1.95% to 41.71% (dry weight) after 2 days of nutrient stress induction. Short-term nutrient-stress treatment had little effect on frond photosynthesis, enhanced the activity of starch synthesis-related enzyme, and weakened the activity of degradation-related enzymes. The transcriptome results further indicated that the key genes and metabolic patterns of starch synthesis promoted starch accumulation in *S. polyrhiza ZH0196* fronds by accelerating the response to CO_2_ fixation via the Calvin cycle, promoting straight-chain starch synthesis, and decreasing starch degradation after short-term oligotrophic treatment. This study suggests that nutrient stress is a green and efficient method of increasing the starch yield of duckweed, which represents an important insight for developing duckweed starch resources.

## 1. Introduction

Duckweed is a small and simple aquatic plant characterized by its rapid reproductive capacity, with the ability to double its biomass in approximately 2–3 days [1]. Under suitable culture conditions, duckweed can produce carbohydrate constituting 38.38–41.68% [2] of its dry weight. This high starch content, combined with minimal lignin and cellulose [3], positions duckweed as a promising candidate for various applications, particularly as a biofuel feedstock [4].

The unique structure and properties of duckweed lend themselves to a diverse range of applications. Its cultivation does not require soil, enabling multilayer cultivation that is environmentally friendly. Additionally, several studies have demonstrated that duckweed effectively purifies wastewater [5,6], as its fronds can absorb heavy metal ions while efficiently enriching nitrogen and phosphorus. The rapid growth of duckweed in nutrient-rich waters also provides a source material for the fermentation of energy substances, such as ethanol [7]. Furthermore, as an edible plant [8], duckweed has been explored for uses in animal feed, vaccine production, and the development of functional components [9]. Meanwhile, duckweed has been increasingly recognized as a new food resource for human nutrition [10]. Therefore, it is very necessary to develop the starch resources of duckweed.

The growth and biomass accumulation of duckweed are influenced by various environmental factors, including light intensity [11,12], spectral composition [13], nutrient solution composition, heavy metal ions [14], plant hormones [15], and temperature. Among these, phosphorus and nitrogen are critical elements in the nutrient solution that support duckweed growth [16]. Interestingly, while deficiencies in nitrogen and phosphorus can inhibit growth, they significantly enhance starch content. For instance, under phosphorus-deficient conditions, the activities of key enzymes such as ADP-glucose pyrophosphorylase (AGPase) and soluble starch synthase (SSS) in *Landoltia punctata* increase, while nitrogen deficiency alters the activity and expression of carbon fixation enzymes [17]. Similarly, sulfur deficiency can lead to increased starch yield without affecting growth [18]. The phenomenon of “nutrient stress”, whereby a lack of all nutrient elements stimulates dry matter accumulation, also results in minimal environmental pollution.

Despite its potential, research on starch synthesis and metabolism in duckweed remains limited compared to starchy grain crops. The field of plant biotechnology faces the challenge of developing high-yield crops to meet the needs of a growing global population. Duckweed’s metabolic plasticity, evidenced by its short growth cycle, small gene pool, and adaptability, presents an opportunity to address starch yield limitations [19]. While 35 species of duckweed belonging to five genera have been identified, significant variations exist in biomass accumulation among different species under varying culture conditions. For example, the genera Lemna, Spirodela, and Wolffia differ in their morphological, physiological, and biochemical characteristics, which affect their response to environmental factors such as nutrients, light, and temperature [20].

Promoting the breeding of starch-accumulating duckweed through transgenic technology is still in its infancy. Starch synthesis in duckweed is analogous to that in other green plants, as it synthesizes organic matter via photosynthesis. The plant absorbs and fixes CO_2_ through the Calvin cycle, ultimately forming starch, which is utilized in cellular metabolism and stored as granules in chloroplasts. Research has shown that, in *Lysimachia nummularia*, the formation of dormant bodies is influenced by ABA, with starch content negatively correlated with α- and β-amylase activities [21]. However, systematic large-scale studies focusing on the mechanisms of starch accumulation in duckweed are scarce, primarily due to genetic differences among various germplasms and inconsistencies in induction conditions. Therefore, further investigation into the biological processes and mechanisms of starch accumulation in duckweed is essential to identify effective methods for regulating its starch production.

This study primarily focuses on two culture conditions that affect biological changes in the starch of duckweed fronds, including the activities of starch synthase and catabolic enzymes. The strain *S. polyrhiza ZH0196* was assessed to determine how nutrient stress conditions influence starch accumulation patterns, starch-related enzyme activity, and transcriptome profiles, elucidating the patterns and mechanisms of starch accumulation. The results will provide a foundation for the development and application of duckweed starch.

## 2. Results

### 2.1. Growth Characteristics in ZH0196 Frond Under Nutrient Stress

#### 2.1.1. Morphological Changes in Fronds Under Nutrient Stress

**Figure 1:**
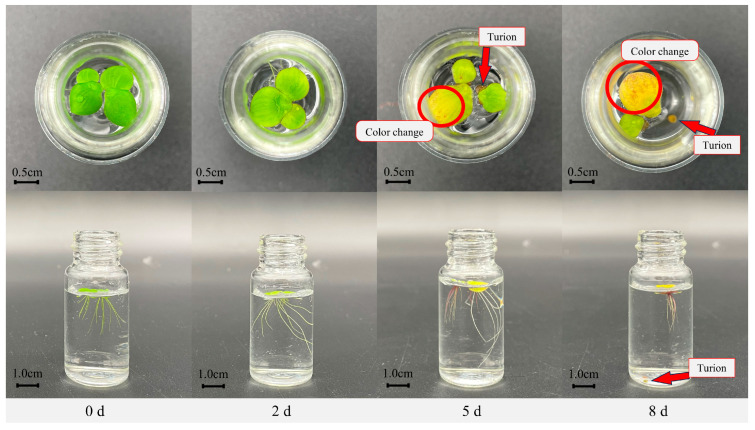
Changes in morphology after nutritional stress on *S. polyrhiza ZH0196*. The red arrows in the image indicate the turions; the red circles indicate areas of color change in the fronds. The nutritional stress culture solution is replaced with deionized water for nutrient stress.

In normal culture conditions (1/5Hoagland), *S. polyrhiza ZH0196* exhibits a green frond appearance, characterized by large, round, or oval-shaped leaves (as shown in Figure 1, 0 d). The propagation of fronds is initiated by the emergence of small new leaves from two neighboring fronds of the same plant. These new leaves gradually grow and detach from the original leaves, thereby forming a new plant body. *ZH0196* has been observed to exhibit rapid growth under conditions that are conducive to its nutrient requirements. However, after nutrient stress treatment, the growth characteristics of *S. polyrhiza ZH0196* changed in frond color, growth speed, root length, and turion. Firstly, the frond color gradually changed. By the second day of nutrient stress treatment, the frond surface (the part above water) remained green but turned yellow in the following days (as circled in Figure 1). The back of the frond gradually changed from green to purple on the fourth day after nutrient stress induction. In the days that followed, all fronds changed color to yellow or white, which means death. Meanwhile, the growth speed of *S. polyrhiza ZH0196* slowed down after short-term nutrient stress treatment, but the dry weight of duckweed increased. The slow-down growth was reflected in the fact that the number of fronds hardly increased. And the roots elongated and tried to obtain more nutrients. However, there is no more N or P in the culture medium. So, *S. polyrhiza ZH0196* reproduction seemed to cease. On the eighth day after induction, roots were detached from the fronds. After induction, a dark green oval was observed between two fronds (as red arrows in Figure 1), which are called turions. When the turions matured, they separated from the fronds and sank to the bottom of the water, which can be seen at the bottom of the bottle.

#### 2.1.2. Starch Granule Changes in Frond Under Nutrient Stress

**Figure 2:**
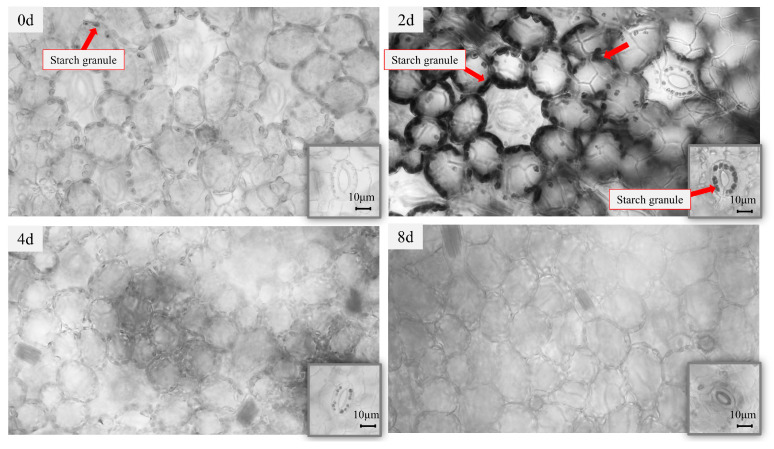
Frond cells of *S. polyrhiza ZH0196* at different induction times under a 40× light microscope. The red arrows in the image also indicate the locations of starch granules. The small figure in the lower right corner of the picture shows the leaf stomatal cells. Starch staining of *ZH0196* fronds by Lugol’s staining method, observed at 40× microscope.

In standard cultivation conditions, the frond starch accumulation of *ZH0196* is depicted in Figure 2 0 d. The accumulation of starch granules in the cells is minimal. Starch accumulation was visualized using light microscopy. Distinctly darkened granules were observed at the edges of the cells after 2 days of nutrient stress, which indicates starch accumulation (as shown in Figure 2). The main cell organelle of starch synthesis is the chloroplast. However, the center of the cell is considered a large vacuole for high (up to 90%) water content in duckweeds. Therefore, other organelles including chloroplasts were squeezed to the edges.

### 2.2. Photosystem II (PS II) Activity

**Figure 3:**
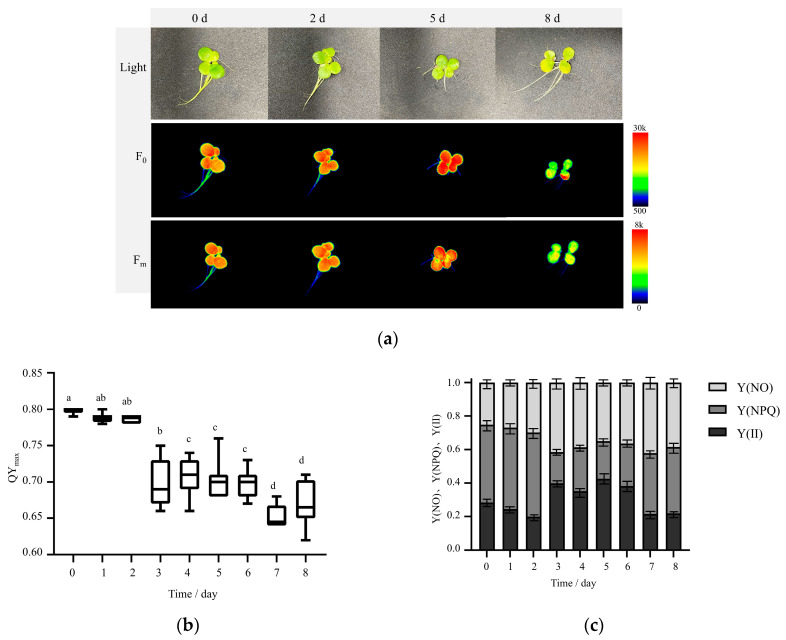
Comparison of chlorophyll fluorescence characteristics and changes in chlorophyll parameters in *S. polyrhiza ZH0196* fronds at different induction times: (**a**) From top to bottom, plant morphology, chlorophyll minimum fluorescence yield F_0_, and maximal fluorescence yield F_m_. Fluorescent bars from red to blue indicate decreasing photosynthetic intensity. (**b**) Maximum photochemical efficiency QY_max_ (F_v_/F_m_), reflects the efficiency of photosynthesis. (**c**) Energy partitioning of Y(II), Y(NPQ), and Y(NO). Y(II) reflects PSII, the actual light energy conversion efficiency of leaves under light. Y (NPQ) refers to the quantum yield of regulated energy dissipation at PS II. Y (NO) refers to the quantum yield of non-regulated energy dissipation at PS II. Different letters indicate significant differences among groups at *p* < 0.05, according to Duncan’s test.

The chlorophyll fluorescence characteristics of *S. polyrhiza ZH0196* were detected and photographed under nondestructive conditions, as shown in Figure 3a. F_0_ is the fluorescence origin. It is the fluorescence yield of the photosystem II (PSII) reaction when the center is fully open, and it is related to the chlorophyll concentration in the leaves. Fm is fluorescence maximal, which is the yield of PSII. When the reaction center is completely closed, it can reflect the electron transmission through PSII. The yellowing of the fronds was observed. The fluorescence intensity of chlorophyll remained high for 2 days of nutrient stress treatment but decreased significantly on day 6, indicating that chlorophyll had been destroyed on the sixth day of nutrient stress treatment. QY_max_ (F_v_/F_m_, Maximum Quantum Yield of Photosystem II Photochemistry) is a parameter representing the function of PSII, which reflects the efficiency of photosynthesis, which is shown in Figure 3b. Calculations of the chlorophyll fluorescence parameters are shown in Figure 3c. Y(II), Y(NPQ), and Y(NO) PSII changed with treatment time. Y (NPQ) decreased significantly in the middle of the treatment (4 d to 6 d), whereas, in the late treatment (8 d and later), however, the Y (NPQ) values showed a rebound.

### 2.3. Starch Yield in Fronds During Nutrient Stress

**Figure 4:**
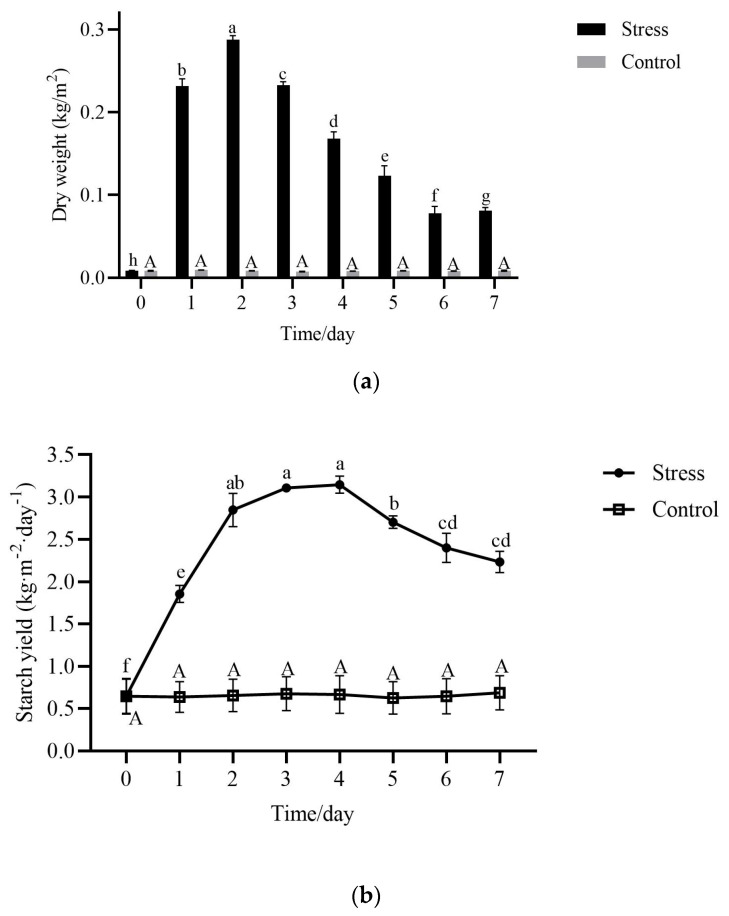
Changes in dry weight and starch yield in *S. polyrhiza ZH0196* fronds within 7 days under nutritional stress: (**a**) Dry weight represents the weight of all duckweed plants in the unit area, represented by a bar graph. (**b**) Starch yield is the average daily starch yield of the duckweed per unit area from the beginning of induction, represented by a line graph. Stress stands for nutrient stress, and control stands for nutrient culture conditions. Uppercase letters for control and lowercase letters for stress. Different letters indicate significant differences among groups at *p* < 0.05, according to Duncan’s test.

The unit area was calculated on the basis of frond spread over the water surface, and the change in dry weight per unit area of the frond of *ZH0196* and the change in starch yield were both found to be negligible under standard culture conditions. As shown in Figure 4, starch yield reached 0.75 kg·m^−2^·day^−1^ on the 2–4th day. It is noteworthy that the results here were in the monolayer culture. And the height required for duckweed cultivation is not large (20–40 cm is sufficient). If multilayer culture is practiced, the starch yield would double with the number of layers. Starch yield depends on starch content and dry weight. The frond dry weight of *S. polyrhiza ZH0196* accumulated, and the starch content of the frond (as shown in Table 1) increased rapidly within 2 days after nutrient stress. The dry weight reached the initial mass by 4.4 times on the second day. Meanwhile, the changes in starch content can also explain the changes in dry weight. The rapid growth of starch leads to a rapid increase in dry weight within 2 days. The starch content reached the highest value on the second day, which increased from 1.95% to 41.71%, representing an increase of 21 times. The starch content gradually decreased after the fourth day, which was due to the transfer of frond starch as the turions grew. Then, with the maturation and shedding of turions, the starch content of the *S. polyrhiza ZH0196* frond began to decrease. Subsequently, dry weight began to decline on the fifth day.

### 2.4. Starch Composition and Accumulation Rate

**Figure 5:**
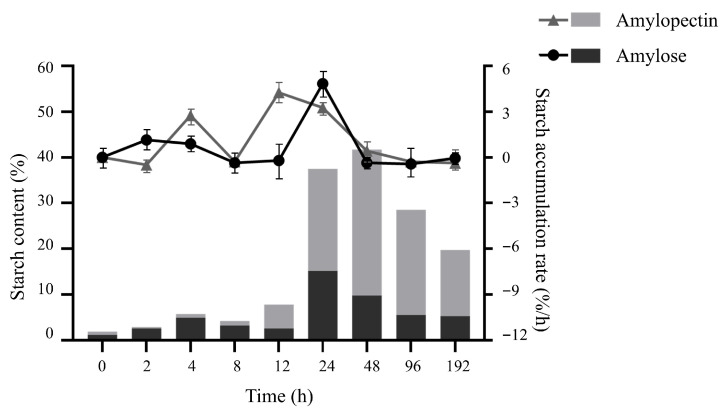
Changes in amylopectin and amylose content and accumulation rate in *S. polyrhiza ZH0196* fronds within 192 h after nutritional stress induction. The column bar chart represents starch content, while the line chart represents the accumulation rate.

Considering that the rate of starch accumulation in duckweed at different time points of induction was different, eight days in the pre- and mid-induction stages were subdivided into 192 h, and nine points were selected for the measurement. After the content of amylose and amylopectin was determined separately, the calculated results are shown in Table 2. It is evident that the highest levels of amylose and amylopectin occur at 24 h and 48 h, respectively, which correspond to the time when the total starch content is high. At the same time, it can also be seen in Figure 5 that the proportion of amylopectin in starch was higher after 24 h of nutrient stress treatment, while the amylose content gradually decreased. Therefore, it is speculated that amylose was synthesized first, and amylopectin accumulates at a later stage in *S. polyrhiza ZH0196* fronds.

In the meantime, the accumulation rates of the two starch contents were also calculated as shown in Table 2. Most accumulation rates of amylose and amylopectin were positive within 24 h, indicating that starch was increased. However, after 48 h, the growth rate of amylose was negative, indicating that amylose accumulation decreased. At the same time, the growth rate of amylopectin decreased to below 0 after 96 h, indicating that the amylopectin content was also decreasing.

### 2.5. Enzyme Activity Related to Starch Metabolism

**Figure 6:**
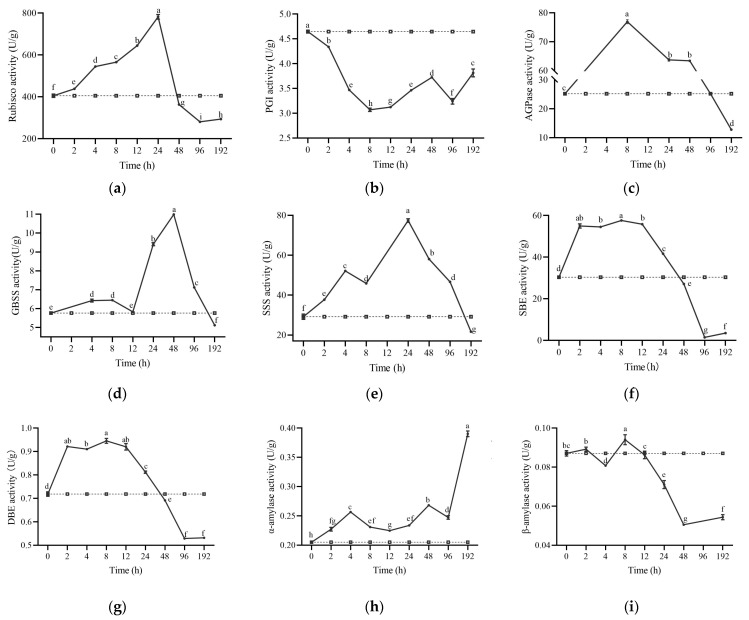
Dynamics of enzyme activities related to starch synthesis at different times of nutrient stress in *S. polyrhiza ZH0196*: (**a**) Rubisco activity. (**b**) Phosphoglucose isomerase (PGI) activity. (**c**) ADP-glucose pyrophosphorylase (AGPase) activity. (**d**) Granule-bound starch synthase (GBSS) activity. (**e**) Soluble Amyl Synthase (SSS) activity. (**f**) Starch Branching Enzyme (SBE) activity. (**g**) Starch Debranching Enzyme (DBE) activity. (**h**) α-Amylase activity. (**i**) β-Amylase activity. The square represents control and the circle represents stress. Different letters indicate significant differences among groups at *p* < 0.05, according to Duncan’s test.

After nutrient stress induction in *S. polyrhiza ZH0196*, the activities of enzymes involved in starch synthesis were enhanced. After stress, GBSS, SSS, SBE, and DBE activity responded and increased in 2 h, as shown in Figure 6. The strongest enzyme activity occurred in 12–48 h after treatment, which belonged to the pre-phase when starch synthesis was accelerated, and starch content increased rapidly. But, after 96 h of treatment, starch synthesis enzyme activity was inhibited. As shown in Figure 6, starch degradation enzymes, such as α-amylase, showed little change in the early stage of induction but increased significantly after 96 h. Similarly, β-amylase activity showed little change in the pre-phase of treatment but decreased significantly after 24 h. Under nutrient stress, CO_2_ fixed by photosynthesis may be the only carbon source for *S. polyrhiza ZH0196*.

Before the starch synthesis reaction occurs, the activity of the enzymes involved in carbon fixation by photosynthesis changes, as shown in Figure 6. Rubisco activity increased after nutrient stress treatment, and the enzyme activity gradually decreased to a level lower than the initial enzyme activity at 48 h of induction, indicating that the rate of CO_2_ fixation accelerated within 48 h of induction. PGI activity after induction was persistently lower than that of the starting value, indicating that the interconversion efficiency between fructose-6-phosphate and glucose-6-phosphate was reduced. AGPase activity increased significantly after induction, remained high until 96 h, and then decreased to lower than the initial value after 192 h.

**Figure 7:**
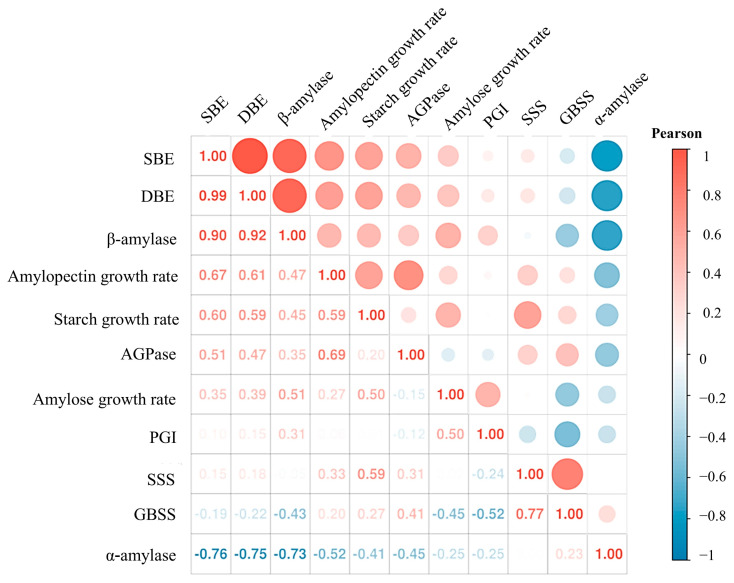
Correlation between starch metabolizing enzyme activity. The Pearson coefficient has a range of values from blue to red indicating −1 to 1, with −1 to 1 representing a stronger correlation, 1 indicating a perfect positive correlation, −1 indicating a perfect negative correlation, and 0 indicating no linear correlation.

In the meantime, the correlation between the activities of different enzymes is shown in Figure 7. DBE and SBE are the most correlated because they both play a role in amylopectin synthesis. It is surprising that β-amylase activity was positively correlated with some starch synthase enzymes, such as DBE and SBE. It is speculated that the reaction principle of β-amylase is to cut the separated α-1,4 bonds sequentially from the non-reducing ends and participate in the synthesis of amylopectin and starch by modifying the starch. In addition, GBSS, AGPase, and SSS had a close relationship after the nutrient stress treatment. They all had a positive effect on amylose synthesis.

### 2.6. Differentially Expressed Gene

**Figure 8:**
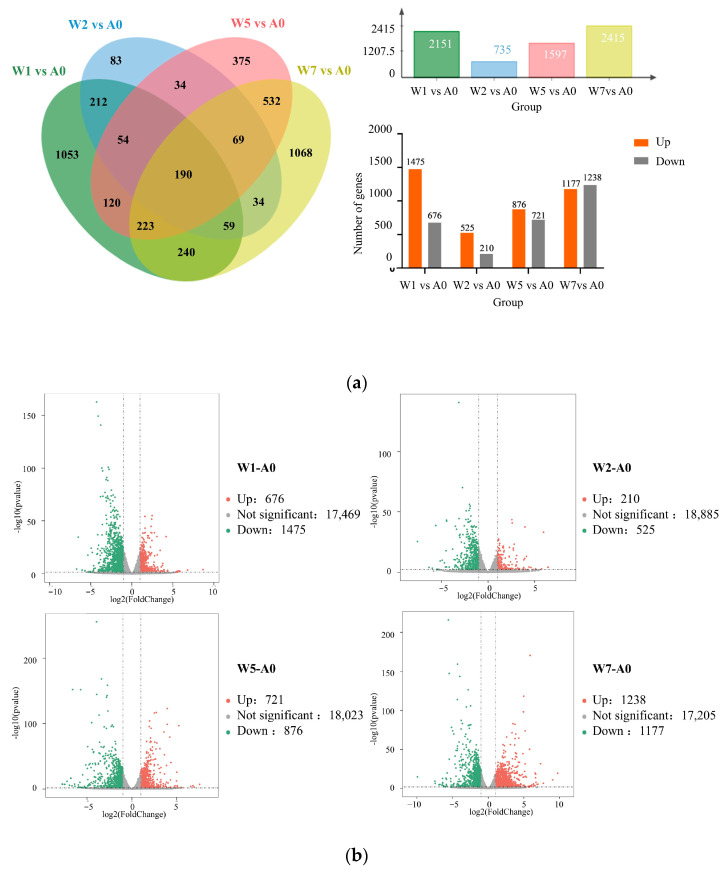
Venn diagram and volcano plots of differentially expressed genes (DEGs): (**a**) Venn diagram of differential genes between groups; (**b**) volcano plots of DEGs, the horizontal coordinate represents log2(fold change), and the vertical coordinate represents the negative logarithmic value of the *p*-value for the change in gene expression. |log2(fold change)| > 1 (as indicated by the dashed line) indicates that there is a significant difference in the expression of the differential genes; red indicates increased expression, green indicates decreased expression, and grey indicates no significant difference.

The number of DEGs and the up/down regulation genes between groups were determined by a statistical analysis of genes screened between sample groups. A total of 4346 DEGs were detected in the fronds during the induction of *S. polyrhiza ZH0196*, among which 190 were co-expressed (Figure 8a). They might play a role in some important pathways. After calculating DEGs between two different time points, the highest number of DEGs overlap was 1014 in group W7−W5. The number for group W2−W1 was 515. It was indicated that the groups of different time points had a large number of gene expression changes after stress, which may point to some core genes or pathways. These DEGs may be involved in the metabolic pathways affecting the growth and development of *S. polyrhiza ZH0196* under starvation stress. In total, 2151, 735, 1597, and 2415 DEGs were identified at 2 h, 4 h, 24 h, and 96 h, respectively.

### 2.7. Gene Ontology Enrichment Analysis

After the induction for 2 h, clear differences were observed at the transcriptome level of *S. polyrhiza ZH0196*. Each point in the graph represents a gene, with the red color indicating an increase in differential expression and the green color indicating a decrease in differential expression (Figure 8b). The genes on the top-right represent the greater the fold change in the DEG and the more significant the difference. After 2 h and after stress treatment, the transcriptome of *S. polyrhiza ZH0196* was quite different, which was shown by the number of DEGs, even more than that at other time points. The abrupt shift from a trophic state to an oligotrophic state requires a large response to changes in the environment. Meanwhile, there were many genes involved in the biological metabolism response that changed significantly. Therefore, the decrease in the number of differential genes in the next 4 h and 24 h may mean that *S. polyrhiza ZH0196* gradually adapts to the environment.

**Figure 9:**
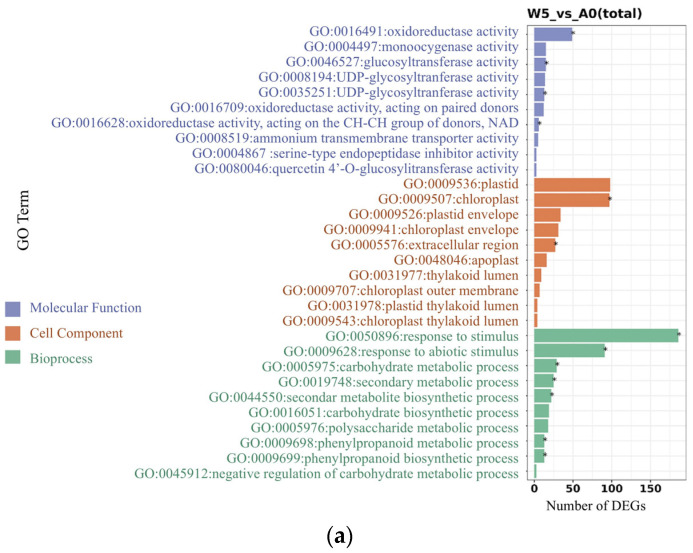

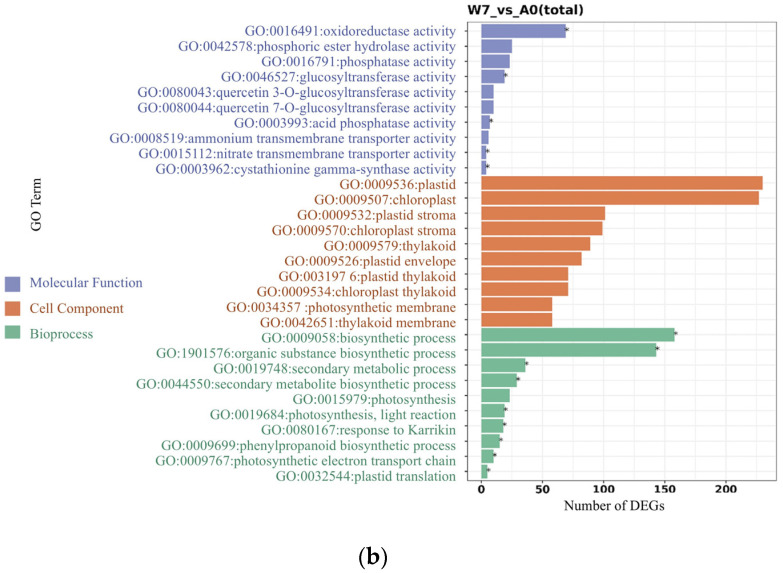
GO enrichment analysis bar graph of differentially expressed genes between groups. (**a**) group W5 and A0; (**b**) group W7 and A0. The vertical coordinate is the GO classification entry that is significantly different (*p*-value), and the horizontal coordinate is the number of differential genes enriched in that GO entry. From the results, the 10 GO terms with the highest number of enrichments were selected to be shown in the bar graph, or all of them if there were fewer than 10 entries (blue—molecular function; red—cell component; and green—bioprocess; *—significant).

As shown in Figure 9, the top ten enriched terms from the taxonomic enrichment analysis are presented, revealing that the GO terms enriched across different comparison groups include oxidoreductase activity under the biological process category, as well as plastids and chloroplasts within the cellular component category. Additionally, terms related to responses to stimuli and carbohydrate metabolism were found in the molecular function category.

### 2.8. Effects of Nutritional Stress on Starch Metabolic Processes

#### 2.8.1. Effects on Starch and Sucrose Metabolism Pathway

**Figure 10:**
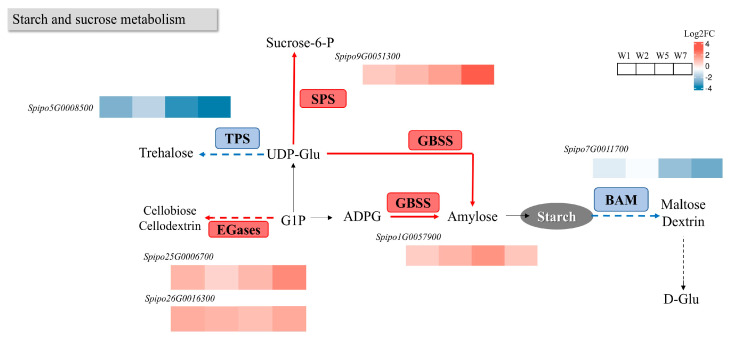
Expression of differentially enriched genes enriched in GBSS, BAM, SPS, TPS of starch and sucrose metabolism pathways compared with A0. Among them, red indicates that gene expression is upregulated, and blue indicates that gene expression is downregulated. The solid line represents the direct reaction process, and the dotted line represents the indirect reaction process. The boxes indicate the enzymes involved in the reaction, including *SPS*: sucrose phosphorylase; *TPS*: trehalose-6-phosphatase; *GBSS*: granule-bound starch synthase; *EGases*: endoglucanase; *BAM*: β-amylase. Black text indicates the reactant or product: Sucrose-6-P: Sucrose-6-phosphate; Trehalose: trehalose; UDP-Glu: uridine diphosphate glucose; G1P: glucose-1-phosphate; and ADPG: adenosine diphosphate glucose. Log2FC from blue to red indicates a log2 (fold change) value from −1 to 1. Enrichment of genes under different stress times in *ZH0196*, their IDs, and expressions are indicated by different color blocks in this figure.

To further elucidate the mechanisms underlying starch accumulation under oligotrophic conditions, we conducted a differential analysis of genes within starch-related KEGG metabolic pathways, with results depicted in Figure 10. Notable changes in gene expression were observed in pathways directly associated with starch anabolism, specifically between the granule-bound starch synthase (*GBSS*) and beta-amylase (*BAM*) pathways. GBSS is primarily responsible for the synthesis of amylose, which joins glucose units together to form a linear structure by catalyzing the formation of α-1,4-glycosidic bonds. The main function of BAM is to hydrolyze starch and its derivatives, releasing reducing sugars that are able to break down starch into smaller sugar units.

Additionally, two genes within the Endo-Glucanase *(EGase*) pathway showed upregulation, while one gene in the Sucrose Phosphate Synthase (*SPS*) and Trehalose Phosphate Synthase (*TPS*) pathway also exhibited differential expression. SPS catalyzes the reaction of sucrose with inorganic phosphate to form glucose-1-phosphate and fructose. SPS provides a source of glucose for starch synthesis. Glucose-1-phosphate is an important intermediate in starch synthesis and can enter the starch synthesis pathway by conversion to ADP-glucose (ADP-Glucose). TPS catalyzes the hydrolysis of trehalose-6-phosphate to produce trehalose and inorganic phosphate (Pi). The activity of TPS can affect the intracellular level of trehalose-6-phosphate, which in turn affects the starch synthesis pathway. EGase breaks down starch and cellulose primarily by hydrolyzing the β-1,4-glycosidic bond, releasing smaller sugar molecules (e.g., glucose and maltose). This hydrolysis allows stored starch to be converted into an available sugar source that provides energy for plant growth and development. Although not directly involved in starch synthesis, it plays a key role in the degradation of starch.

#### 2.8.2. Effects on Carbon Fixation in Photosynthetic Organism Pathway

**Figure 11:**
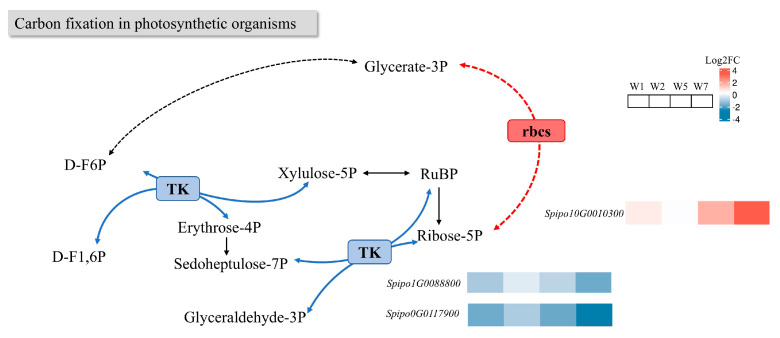
Carbon fixation in photosynthetic organisms involves pathway enrichment and differential gene expression compared to A0. Among them, red indicates that gene expression is upregulated, and blue indicates that gene expression is downregulated. The solid line represents the direct reaction process, and the dotted line represents the indirect reaction process. The boxes indicate the enzymes involved in the reaction, including *TK*: *transketolase*; *rbcs*: *subunit regulating ribulose-1,5-bisphosphate carboxylase/oxygenase*; RuBP: ribulose diphosphate; D-F6P: fructose 6-phosphate; and D-F1,6P: fructose 1,6-bisphosphate. Log2FC from blue to red indicates a log2 (fold change) value from −1 to 1. Enrichment of genes under different stress times in *ZH0196*, their IDs, and expressions are indicated by different color blocks in this figure.

As shown in Figure 11, *rbcs* is involved in the synthesis of Rubisco, which catalyzes the rate-limiting step in carbon fixation [22]. Considerable research has focused on adapting Rubisco to improve light efficiency. Post-induction, the upregulation of *rbcs* in *S. polyrhiza ZH0196* indicates an acceleration of Rubisco enzyme activity, which is consistent with previous measurements of Rubisco activity. Transketolase (*TK*) participates in various metabolic processes within the pentose phosphate pathway. Reduced *TK* activity has been linked to diminished growth, yield, and stress tolerance in cucumber plants [23]. The downregulation of *TK* in *S. polyrhiza ZH0196* following induction may reflect decreased tolerance associated with nutrient deficiencies, adversely affecting growth and yield.

## 3. Discussion

Under optimal conditions, the fronds of *ZH0196* primarily utilize photosynthetic products for growth and reproduction. When daily nutrient supplementation is provided, the fronds are well nourished; despite an increase in the number of fronds, the dry weight per unit area—calculated based on the area of fronds spread over a single layer of water surface—remains relatively unchanged [24]. However, in nutrient-poor environments, the growth of *S. polyrhiza* is inhibited. In such conditions, carbohydrates produced through photosynthesis are no longer utilized for rapid growth; instead, they are increasingly converted into starch for storage as a response to unfavorable circumstances. Similar findings have been reported in other studies. For instance, *Lemna aequinoctialis* exhibited a starch content that increased to 35% of its dry weight under low-nutrient and high-light conditions, representing a two-fold enhancement compared to standard conditions [25]. *Landoltia punctata* cultured in nitrogen- and phosphorus-free medium can exhibit a significant increase in starch content, rising from a basal level of approximately 3% to over 50.33% within just 7 days [11,26]. When essential nutrients such as nitrogen, phosphorus, or sulfur are deficient, plant growth is suppressed; however, photosynthesis continues to occur to some degree, leading to an accumulation of excess carbohydrates within the plant [27,28]. These excess carbohydrates are subsequently converted into starch and stored [29]. This phenomenon may also account for the increased starch production and dry weight observed in *ZH0196* following nutrient stress.

The accumulation of starch was similar to the experimental results obtained under a lack of phosphorus [30] or nitrogen [17], through the different duckweed species. When the water is nutrient-rich and the temperature and light conditions are suitable, duckweed continuously reproduces at a high rate [31], and nothing can stop the plant from growing [32]. And when the culture environment was adjusted (e.g., nutrient changes, hormone induction, and temperature increases or decreases), the growth and development patterns differed. The fronds would be starch-rich under certain conditions [4,33]. However, the starch growth process of *S. polyrhiza ZH0196* frond is transient and would be transferred to producing turion. At the second hour after induction, the amylose content and the proportion of straight-chain starch in *ZH0196* chloroplasts reached the peak, and the proportion of branched-chain starch was the lowest, which might be the rapid response of *ZH0196* to the nutrient stress conditions, and it mainly synthesized straight-chain starch to cope with nutrient deficiencies. At the 24th hour, amylose content in the chloroplasts of *ZH0196* chloroplasts was increased again, the proportion of straight-chain starch decreased, and the proportion of branched-chain starch increased, which indicated that chloroplasts might start to switch to storing and using branched-chain starch as an energy source in this stage. This suggests that the chloroplasts may be shifting to storing and utilizing branched-chain starch as an energy source at this stage.

We divided the nutrient stress period of *S. polyrhiza ZH0196* into three stages: pre-, mid-, and post-phases, which were based on the changes in growth. In the pre-phase (usually 0–4th d), growth speed slowed, but starch accumulated rapidly in *S. polyrhiza* fronds. In the mid-phase (4–8th d), the frond color gradually turned yellow, and the starch in fronds gradually decreased. In the meantime, frond starch was shifted to the turions for surviving. In the post-phase (8th d to death), no starch appeared to be stained in the fronds, which means all starch in the fronds was transferred or consumed until death. However, this division time is not absolute and will be affected by duckweed species, water surface coverage, light intensity, and other conditions.

The QY_max_ value of optimum is 0.83, and 0.60 is relatively low, but probably not low enough to indicate a significant stress [34]. When plants are subjected to stress, such as drought [35] and salt stress [36], QY_max_ decreases significantly. On the 4th day after induction, QY_max_ was significantly lower than normal, indicating that photosynthesis was inhibited. In the meantime, the photochemical quenching of NPQ began to decrease significantly, implying that the PSII system was damaged. In the post-phase (8th day and later), NPQ rebounded owing to the weakening of photosynthesis and chlorophyll fluorescence in the PSII system, and the light energy received by the plant was emitted as heat energy for self-preservation. Y(NO) is the quantum yield of unregulated energy dissipation at PS II, which is an important indicator of photodamage. After the 3rd day of induction, Y(NO) increased, indicating that the photochemical energy conversion and protective regulatory mechanisms were not enough to completely consume the light energy absorbed by the plant, which may indicate that the frond has been damaged.

GBSS is related to the synthesis of amylose [37] and catalyzes the formation of amylose by transferring glucose molecules to ADPG (starch precursors). The synthesis of amylopectin is accomplished by three enzymes: SSS is extended by an α-1,4 glycosidic linkage to glucose, a 1,6 glycosidic bond is formed by the SBE to produce branching, and then the DBE removes the unsuitable bonds to form a helical structure that is easy to crystallize. Starch in the leaves is stored in the early stages of induction, begins to degrade in the later stage, and is then used for energy or transferred. The content of amylose and amylopectin is the main factor affecting the yield and quality of starch, which are produced from these four different starch synthases in plants. However, Rubisco, PGI, and AGPase, which are involved in photosynthesis, affect starch synthesis and play crucial roles in carbon assimilation and starch synthesis. Rubisco is responsible for the fixation of almost all atmospheric CO_2_ [38], catalyzes reactions between CO_2_ and ribulose 1,5-bisphosphate (RuBP), and is the initial enzyme in the carbon fixation phase of the Calvin cycle. The role of PGI in chloroplasts is to link the Calvin cycle with the starch biosynthesis pathway [39] and catalyze the interconversion of glucose-6-phosphate (G6P) and fructose 6-phosphate (F6P) [40]. AGPase catalyzes the reaction of G1P with ATP to generate ADPG (direct precursor starch), which is generally considered the rate-limiting step in starch synthesis during photosynthesis. Studies [41] have shown that the lack of cPGI activity in leaves can lead to excessive starch accumulation in plants, and experiments have confirmed that the reason for this may be a reduction in intracellular glucose turnover and chloroplast starch degradation. Increased AGPase activity is typically a sign of accelerated starch synthesis or starch accumulation [42].

Chlorophyll fluorescence is correlated with the Rubisco concentration [43], and the above results suggest that, from 0 to 48 h in the pre-phase, chloroplast functions were normal, and photosynthesis efficiency was basically unchanged. Thus, photosynthesis was functioning normally. Nutrient stress treatment promoted CO_2_ fixation in *S. polyrhiza ZH0196* fronds; however, the conversion efficiency of F6P and G6P decreased, thus providing buffer time for ADPG synthesis. Simultaneously, the activity of AGPase significantly increased, and the accumulation of starch precursors increased, resulting in starch accumulation in the chloroplasts. In the mid- and post-phases of induction (6–8th d), chlorophyll was destroyed, chloroplast functions were damaged, photosynthetic efficiency decreased significantly, CO_2_ could not be fixed normally, and starch could not be synthesized via photosynthesis. Starch that has already been synthesized was then broken down for plant growth energy or transferred, resulting in a decrease in starch content.

The expression levels of certain functional genes involved in regulation gradually returned to baseline levels. The specific categories of differentially expressed genes (DEGs) were identified through Gene Ontology (GO) analysis. Notably, the number of downregulated genes among the DEGs exceeded that of upregulated genes during the first three time points, likely due to the adaptation to adverse environmental conditions. In response to nutrient deficiencies, *S. polyrhiza ZH0196* may selectively downregulate specific genes to conserve energy and resources, reflecting its strategy for survival under challenging conditions. Furthermore, the downregulation of genes may also correlate with the inhibition of certain biological processes. GO enrichment analyses provide insights into gene functions based on the distribution of DEGs [44]. The transcriptome exhibited enrichment in the specified GO classification terms. These enrichment terms include oxidoreductase activity under the biological process category, as well as plastids and chloroplasts in the cellular component category. The observed number of DEGs is likely linked to the capacity of oligotrophic acid to stimulate the growth of fronds in nutrient solutions, resulting in oxidative stress. Consequently, oxidoreductase activity is essential for scavenging free radicals generated by these reactions, thereby mitigating damage to *S. polyrhiza ZH0196*. In addition, in the category of molecular function, we found associations with stimulus responses and carbohydrate metabolism. These organisms may have evolved to enhance carbohydrate synthesis and metabolism as an adaptation to oligotrophic conditions, thereby altering biochemical reactions associated with starch formation and storage in the chloroplast matrix, thylakoids, plastids, and other cellular components. These findings elucidate the effects of oligotrophic culture on the transcriptome and the corresponding changes in biological processes, cellular components, and carbohydrate synthesis.

The expression of the *GBSS* gene was significantly upregulated, leading to an accelerated synthesis of amylose and correlating with increased *GBSS* enzyme activity, which is a key regulator of amylose production [45]. *GBSS* encodes Granulose Starch-Binding Enzyme I (*GBSSI*), which specifically elongates starch chains [46]. CRISPR/Cas9-mediated editing of the *GBSS* gene has been shown to modulate amylose content, thereby affecting the waxy characteristics of corn starch [47,48]. As starch synthesis accelerated, starch degradation was inhibited. During oligotrophic induction, *BAM* gene expression was significantly downregulated, indicating a reduction in leaf β-amylase activity, which aligns with previous measurements. The *BAM* gene encodes β-amylase, responsible for cleaving α-1,4 glucosidic bonds from the non-reducing ends of polydextran chains to produce maltose [49]. It has been established that KCl is essential for the normal expression of *BAM2* [50], and oligotrophic-induced water production may lack sufficient ions for its utilization, potentially explaining the downregulation of this gene.

While starch accumulated in the leaves, we also observed differentially expressed genes (DEGs) in other pathways related to carbon allocation. The *SPS* gene was significantly upregulated, suggesting an enhancement in the sucrose synthesis pathway. Previous studies have indicated that enzymes involved in sucrose synthesis in *Arabidopsis thaliana* leaves do not participate in starch synthesis [51]. In crops such as rice, sucrose synthesis plays a critical role in transporting photosynthetically fixed carbon from leaves to stems and ultimately to grains, thereby completing the source-to-reservoir transport pathway [52]. In the case of *P. multiflora*, hypnozoites may serve as the ultimate carbon “reservoir”; consequently, while starch accumulates in leaves, some carbon-derived sucrose may also be directed to the hypnozoites. In addition, we noted that *TPS* gene expression was downregulated. Research has shown that *TPS* not only contributes to carbon allocation [53] but also participates in the synthesis of trehalose, a stress metabolite that helps plants mitigate environmental stress. The upregulation of two *EGase*-related cellulase genes was also observed. *EGases* catalyze the hydrolysis of polysaccharides with a 1,4-β-glucan backbone, such as cellulose and xyloglucan, thus playing a role in cell wall modification. Following induction, cellulose decomposition likely occurs, allowing sugars to be synthesized through alternative metabolic pathways, thus enabling duckweed to adapt to stress.

In plants, *rbcs* may be involved in the synthesis of Rubisco, the rate-limiting step in Rubisco-catalyzed carbon fixation. The upregulation of *rbcs* in *ZH0196* after induction indicates an acceleration of Rubisco enzyme activity, which is consistent with previous measurements of Rubisco activity. *TK* is involved in various metabolic processes within the pentose phosphate pathway, and reduced *TK* activity is associated with reduced plant growth, yield, and stress tolerance. The downregulation of *TK* gene expression in *ZH0196* after induction may reflect reduced tolerance associated with nutrient deficiency, which adversely affects growth and yield.

**Figure 12:**
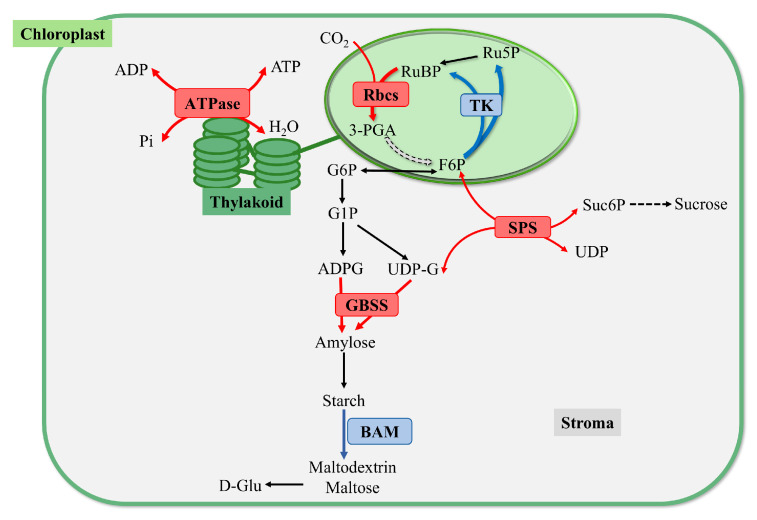
Metabolic pathways for starch synthesis and starch degradation through photosynthesis in chloroplasts in the early stage of nutrient stress. The red arrows indicate the upregulated pathway, and the blue arrows indicate the downregulated pathway. The solid line represents the direct reaction process, and the dotted line represents the indirect reaction process. Double-headed arrows indicate that the reaction is reversible.

In summary (as illustrated in Figure 12), the upregulation of the *rbcs* gene expression in the leaves of *S. polyrhiza* under oligotrophic conditions enhances carbon fixation by facilitating the reaction that fixes CO_2_ in the Calvin cycle. Moreover, the *ATPase* located in the membrane of the quasi-saccule provides sufficient ATP for photosynthesis. In the carbohydrate synthesis metabolic pathway, the upregulation of the *GBSS* gene expression accelerates the synthesis of straight-chain starch, thereby promoting starch accumulation. Conversely, the downregulation of the *BAM* gene inhibits starch degradation, resulting in increased starch content in the leaves.

The starch accumulation response of *S. polyrhiza ZH0196* is linked to energy storage, survival strategies, and developmental changes. Energy storage occurs during the initial phase (0–4 days) when leaves rapidly accumulate starch despite reduced growth. This indicates a response to unfavorable conditions by converting carbon sources into starch for future energy use. As environmental conditions worsen, starch is transferred to turions for survival. The plant adapts to nutrient deficiencies by adjusting starch synthesis and degradation. Accumulated starch serves as an energy reserve and helps maintain viability during adversity, allowing growth and development through internal reserves. Under nutrient stress, *S. polyrhiza ZH0196* modifies its growth patterns, especially when producing winter buds. Turion formation is a key strategy for coping with unfavorable conditions, enabling regrowth when the environment improves. Starch accumulation supports the energy needs for winter bud formation and future reproduction. Overall, the starch accumulation response enhances the plant’s ability to survive challenges and adapt to environmental stressors.

## 4. Materials and Methods

### 4.1. Duckweed Species and Culture Conditions

*S. polyrhiza ZH0196* was obtained from a duckweed germplasm bank (Chengdu, China). The duckweed samples were placed in sterile Hoagland medium containing 1.5% sucrose for activation culture. After covering the water surface with duckweed, open culture was carried out in 1/5 Hoagland medium, with more than 70% coverage maintained for expansion. The culture solution was regularly replenished or changed to ensure sufficient nutrition for the duckweed. When the duckweed had grown to a sufficient size, it was removed from the nutrient solution and rinsed gently with deionized water. We obtained deionized water for the experiments from our laboratory’s deionization system (QinYuan QR-RF-04D, Ningbo, China). The culture solution was then replaced with deionized water for nutrient stress. Nutrient stress was maintained for 8 days. The room temperature was maintained at 25 ± 1 °C, and full white light at an intensity of 2000–4000 LX was applied for 24 h culture every day.

### 4.2. Photosystem II (PS II) Activity Measurement

Three to five *ZH0196* were randomly selected for the experiment. The assayed fronds were fully dark-adapted for about 5 min; then, the fronds were briefly and rapidly illuminated, and changes in fluorescence signals during days 0–8 were detected. The shutter speed was set to Shutter = 0, the sensitivity was set to Sensitivity = 20, the illumination was set to Act2 = 100, Act1 = 100, and Super = 70. The fluorescence parameters were measured, including the minimum fluorescence intensity (F0), the maximum fluorescence intensity (Fm), and the variable fluorescence (Fv). The following were calculated: PSII, maximum photosynthetic efficiency [QYmax (Fv/Fm)], the actual quantum yield under stable illumination [Y(II)], quantum yield of regulated thermal energy dissipation at steady-state [Y(NPQ)], and quantum yield of unregulated energy dissipation [Y(NO)]. A total of 3–5 duckweed plants were randomly selected from each treatment group for determination. The experiment was conducted using the FluorCam 800MF closed plant fluorescence imaging system (PSI, Drásov, Czech Republic) for measurement. Other parameters and treatment methods refer to the experiments conducted by Maxwell et al. [54].

QYmax = Fv ÷ Fm(1)

### 4.3. Growth Measurement

Duckweed washed with residual nutrient solution was transferred to deionized water for induction. Each pot was equipped with 500 ± 10 duckweed plants of similar size to ensure that most of the water surface was covered, but space was still available for new duckweed. Moreover, not all duckweed fronds were covered. Duckweed fronds (or fronds and turions) were harvested simultaneously on 0–8th days, and the dry weight was measured after drying in oven at 55 °C. Growth of *ZH0196* is indicated by the mass of *ZH0196* follicles within the area. Mass indicates dry weight after drying. Three parallel measurements were performed for each treatment duration. For the same incubation time, *ZH0196* fronds under nutrient culture conditions were used as control group, and nutrient stress condition was used as experimental group and expressed as “stress”.

### 4.4. Determination and Yield of the Total Starch Content

The dried duckweed was crushed and passed through a 0.2 mm sieve to obtain whole duckweed powder. The total starch content was determined using the total starch assay kit (Megazyme, Neogen, USA) according to the manufacturer’s instructions. The area was calculated according to the area of frond exactly spread over one layer of water surface. Starch yield is calculated according to the following formula:

Starch yield (g/area) = Content (%) × Dry weight (g)(2)

Starch growth ratio = ∆Starch content ÷ Starch content at previous time(3)

### 4.5. Determination and Calculation of Growth Rate of Amylose/Amylopectin Content

The determination of amylose content was carried out using the amylose detection kit (Megazyme, Neogen, USA), and the determination method used referred to the instructions. The numeric value of subtraction between total starch content and amylose is duckweed amylopectin content. The area was calculated according to the area of frond exactly spread over one layer of water surface. The duckweed amylose/amylopectin growth rate is calculated according to the following formula:

Starch accumulation rate (g/area) = Increment (%) ÷ time (h)(4)

### 4.6. Light Microscopy Starch Observations

Duckweed was randomly sampled at different time points, soaked in 80% (*v*/*v*) ethanol, heated in a water bath at 50 °C, and shaken for 60 min for decolorization. It was then washed twice with deionized water. The frond was stained in 5% (*v*/*v*) Lugol’s solution for 2 min, and the excess staining solution was washed with deionized water. The cells were observed under the light microscope (BX53, Olympus, Japan).

### 4.7. Determination of Starch Metabolism and Photosynthesis-Related Enzyme Activities

Samples were collected at 0 h, 2 h, 4 h, 8 h, 12 h, 24 h, 2 d, 4 d, and 8 d after induction. Plants were randomly sampled from the same culture pot, and three replicates were performed for each sample. After drying the water on the surface of the floating plants, the plant material was quickly cooled in liquid nitrogen and stored in the refrigerator at −80 °C for subsequent enzyme activity measurement and RNA sequencing. After grinding, 0.1 g of the sample was placed in 1 mL of distilled water, homogenized, and centrifuged at 8000 rpm for 10 min. The supernatant was then collected. The corresponding enzymes were measured using Ribulose-1,5-bisphosphate carboxylase/oxygenase (Rubisco), ADP-glucose pyrophosphorylase (ADPase), starch debranching enzyme (DBE), granule-bound starch synthase (GBSS), starch branching enzyme (SBE), and SSS activity kits (all kits were obtained from Solarbio, Beijing, China). The activities of α- and β-amylase were determined using the standard GB/T 5521-2008 (Inspection of grain and oils—Determination of alpha-amylase activity in cereal and cereal products—Colorimetric method). Phosphoglucose isomerase (PGI) activity was measured using an ELISA kit according to the manufacturer’s instructions.

### 4.8. Total RNA Isolation, cDNA Library Construction, and RNA Sequencing

The duckweed fronds at 0 h, 2 h, 4 h, 24 h, and 96 h after inducing nutritional stress were selected for RNA sequencing and named A0, W1, W2, W5, and W7, respectively. One gram of the fronds was collected, and three samples were randomly collected. After extracting total RNA, the mRNA was enriched by polyA using oligo(dT) magnetic beads and randomly interrupted by adding fragmentation buffer for fragmentation. cDNA synthesis was completed under the action of reverse transcriptase, and RNA was obtained by end repair and A base addition. The concentration and purity of the extracted RNA were measured using a NanoDrop 2000, and RNA integrity was assessed by agarose gel electrophoresis. Sequencing libraries were then constructed. RNA extraction and sequencing were performed by BIOSANSHU Co., Ltd. (Nantong, China).

### 4.9. Differential Gene Expression and Enrichment Analysis

Quality control of raw data was performed using fastp software (v0.23.4) to obtain high-quality sequencing data (clean data). Clean data were mapped to a reference genome with *S. polyrhiza* 9509 (https://db.cngb.org/search/project/PRJEB28272/, accessed on 22 May 2025) using STAR software (2.7.11b). The number of fragments per kilobase length (FPKM) from one gene per million reads was used to compare the estimated gene expression between the different genes and different experiments. Differentially expressed genes (DEGs) between the two samples were selected using the following criteria: genes in different groups |log2 (FoldChange)| ≥ 1, with a false discovery rate (FDR) of <0.05. Gene Ontology (GO) enrichment and Kyoto Encyclopedia of Genes and Genomes (KEGG) pathway analyses of DEGs were performed using clusterProfiler.

### 4.10. Statistical Analysis

Excel 2010 software was used for data collation and charting, and SPSS 26.0 software was used for the statistical analysis. Data and graphs were processed using GraphPad Prism 6.0, PowerPoint 2010, and website (https://omic.sanshugroup.com, accessed on 20 May 2025). Differences between treatments were analyzed by one-way analysis of variance (ANOVA) combined with Duncan’s multiple range test, and significance was set at *p* < 0.05.

## 5. Conclusions

In the present study, we observed that, following short-term (0–96 h) nutrient stress treatment, the fronds of *S. polyrhiza ZH0196* exhibited a significant accumulation of starch, while photosynthesis remained relatively unaffected. Enzyme activity analyses revealed that most enzymes associated with starch synthesis and photosynthesis demonstrated increased activity during the stress induction period, whereas enzymes related to starch degradation exhibited reduced activity. Transcriptomic analyses further indicated that key genes and metabolic pathways involved in starch synthesis were upregulated. These changes facilitated starch accumulation in the fronds of *S. polyrhiza ZH0196* by enhancing CO2 fixation through the Calvin cycle, promoting amylose synthesis, and inhibiting starch degradation following short-term nutrient stress treatment. In conclusion, the findings of this study provide valuable insights into the mechanisms underlying starch accumulation in duckweed and hold significant implications for the development and utilization of duckweed starch resources.

## Figures and Tables

**Table 1:** The content and growth ratio of *S. polyrhiza ZH0196* starch within 6 days after nutritional stress induction (different letters indicate significant differences among groups at *p* < 0.05, according to Duncan’s test).

	Day 0	Day 1	Day 2	Day 3	Day 4	Day 5	Day 6
Starch content/%	1.95 ± 0.05 ^e^	37.54 ± 0.86 ^c^	41.71 ± 0.51 ^a^	39.18 ± 0.38 ^b^	40.18 ± 0.81 ^a^	39.06 ± 1.19 ^ab^	33.77 ± 0.81 ^d^
Starch growth ratio	-	18.28	0.11	−0.06	0.03	−0.03	−0.12

**Table 2:** The starch content, amylose, and amylopectin content in the total starch of fronds within 192 h after nutritional stress induction. The content of amylose and amylopectin is the proportion of the total starch. Different letters indicate significant differences among groups at *p* < 0.05, according to Duncan’s test.

	0 h	2 h	4 h	8 h	12 h	24 h	48 h	96 h	192 h
Starch Content/%	1.95 ± 0.05 i	2.95 ± 0.13 h	5.78 ± 0.04 f	4.26 ± 0.06 g	7.84 ± 0.10 e	37.55 ± 0.87 a	41.71 ± 0.51 b	28.58 ± 0.44 c	19.78 ± 0.62 d
Amylose Content in Total Starch/%	63.37 ± 3.12 c	89.65 ± 3.03 a	77.39 ± 4.41 b	76.62 ± 3.79 b	33.30 ± 0.82 e	40.60 ± 1.12 d	23.50 ± 3.84 fg	19.50 ± 2.55 g	26.90 ± 2.61 f
Amylopectin Content in Total Starch/%	36.63 ± 3.12 e	10.34 ± 3.03 g	22.61 ± 4.40 f	23.37 ± 3.79 f	66.68 ± 0.82 c	59.40 ± 1.12 d	76.51 ± 3.84 ab	80.49 ± 2.54 a	73.37 ± 2.61 b

## Data Availability

The RNA-seq raw datasets generated during the current study were deposited in NCBI repository. The datasets supporting the conclusions of this article are included within this article and its additional files.

## Abbreviations

The following abbreviations are used in this manuscript:

ADPG	Adenosine 5′-Diphosphoglucose
AGPase	ADP-glucose pyrophosphorylase
*BAM*	*Arabidopsis β-amylase*
DBE	Starch debranching enzyme
DEGs	Differentially expressed genes
*EGase*	*Endo-1,4-β-D-glucanohydrolase*
F_0_	Minimal Fluorescence
F6P	Fructose-6-phosphate
F_m_	Maximal fluorescence
F_v_	Variable fluorescence
G1P	Glucose 1-phosphate
G6P	Glucose 6-phosphate
GBSS	Granule-bound starch synthase
*GBSS*	*Granule-bound Starch Synthase*
PGI	Phosphoglucose isomerase
PSII	Photosystem II
QY_max_	Maximum Quantum Yield of Photosystem II Photochemistry
*rbcs*	*Small Subunits of Ribulose1,5-bisphosphate Carboxylase/oxygenase*
Rubisco	Ribulose-1,5-bisphosphate carboxylase/oxygenase
RuBP	Ribulose 1,5-bisphosphate
SBE	Starch branching enzyme
*SPS*	*Sucrose Phosphate Synthase*
*S. polyrhiza*	*Spirodela polyrhiza*
SSS	Starch synthase enzyme
*TK*	*Transketolase*
*TPS*	*Trehalose-6-phosphate synthase*
